# A Comparative Study of Piperacillin-Tazobactam With and Without Vancomycin as Empirical Therapy for Febrile Neutropenic Patients With Solid Tumor Malignancies

**DOI:** 10.14740/wjon867w

**Published:** 2015-02-14

**Authors:** Mansoor Sirkhazi, Azmi Sarriff, Noorizan Abd Aziz, Fatma Almana, Osama Arafat, Mahmoud Shorman

**Affiliations:** a Department of Pharmacy, King Fahad Specialist Hospital, Dammam, Saudi Arabia; b School of Pharmaceutical Sciences, University Sains Malaysia, 11800 Minden, Penang, Malaysia; c Faculty of Pharmacy, University Technologia MARA, Selangor DE, Malaysia; d Infection Control Department, King Fahad Specialist Hospital, Dammam, Saudi Arabia; e Internal Medicine Department, Marshall University, Huntington, WV, USA

**Keywords:** Febrile neutropenia, Toxicities, Hematological malignancies, Piperacillin-tazobactam, Vancomycin

## Abstract

**Background:**

The objective of this study was to evaluate the outcomes, mortality and toxicity associated with piperacillin-tazobactam (PT) and the addition of vancomycin (VM) to the empirical treatment of febrile neutropenic cancer patients.

**Method:**

A retrospective study on adult febrile neutropenic patients who were admitted between September 2008 and May 2013 with solid tumor malignancies was conducted at King Fahad Specialist Hospital Dammam, Saudi Arabia.

**Results:**

Out of 86 febrile neutropenic patients, 60 patients were treated with PT group and 26 patients were with PT + VM group. The two groups were comparable in terms of outcome, mortality, nephrotoxicities and hepatotoxicities. The median duration of neutropenia in PT treatment group was 4 days (range 1 - 10) in the female and 7 days (range 1 - 13) in males while in PT + VM 6 days (range 1 - 5) in female and 7.5 days (range 1 - 6) in male with significance P = 0.007. There was no significant difference in terms of duration of fever and length of stay between the two treatment groups. There were no deaths reported during treatment in both groups. In PT, the microbial eradication was 27/40 (67.5%) patients (14/27 (51.9%) of female and 13/27 (48.1%) of male)), whereas it was 13/40 (32.5%) patients (9/13 (69.2%) of female and 4/13 (30.8%) of male)) in PT + VM group. Overall, there was no significant difference in terms of microbiological eradication between the two groups (OR: 1.22; 95% CI: 0.486 - 3.072; X^2^ stat: 0.182; P = 0.67). Response to therapy in clinically defined infections was higher 16/23 (69.56%) in PT treatment group than 7/23 (30.44%) in PT + VM group. But there was no significant difference between the two treatment groups in terms of clinically defined infections (OR: 1.013; 95% CI: 0.359 - 2.862; X^2^ stat: 0.001; P = 0.98). There was no significant difference in renal and liver functions between the two groups in terms of serum creatinine level and clearance, alkaline phosphate and alanine tranferase and gama glutamyl tranferase. The most commonly isolated organisms were *Escherichais coli* (eighteen isolates), *Staphylococcus aureus* (seven isolates), *Streptococcus spp* (six isolates) and *Klebsiella pneumonia* (four isolates). The overall success rate was similar in both treatment arms and treatment was well tolerated, with no severe adverse reactions reported.

**Conclusion:**

Although the addition of VM might provide an additional value for coverage of gram-positive pathogens. This study demonstrates that there was no significant difference in terms of response rate in both treatment groups, which could be due to the low local methicillin-resistant *Staphylococcus aureus* (MRSA) rates and other resistant gram-positive organisms at our institution, stressing the importance of local antibiograms in developing empirical neutropenic fever protocols.

## Introduction

Despite extensive clinical studies, none had recommended a single empirical therapy for the initial treatment of febrile neutropenic patients. The information from previous studies are outdated due to the tremendous changes in the bacterial etiology, sensitivity and resistance patterns as well as the criteria used to assess the treatment outcome. The effectiveness of antibiotic therapy may be influenced by the local patterns of bacterial infections and susceptibility [1, 2]. By tradition, the broad-spectrum antimicrobial agents were used either as mono or combination therapy for empirical treatment in febrile neutropenic cancer patients. Many studies showed 50-70% of response rates with the empirical therapy [3]. It has been suggested that an appropriate empirical administration of broad-spectrum antibiotics might decrease the mortality rate caused by bacterial infections in febrile neutropenia [1]. In this regard, empiric monotherapy of an antipseudomonal β-lactam agent, such as piperacillin-tazobactam (PT), carbapenems or an anti-pseudomonal cephalosporins was recommended as initial management of febrile neutropenia, particularly for the high-risk patients. The addition of vancomycin (VM) to beta-lactam antibiotics as initial therapy is recommended for the management of infectious complications (e.g. hypotension and pneumonia) or if gram-positive microorganism resistance is suspected or proven. Glycopeptide antibiotics are recommended for specific conditions, including suspected catheter-related infection, skin and soft tissue infection, pneumonia, or hemodynamic instability and where the gram-positive organisms are predominant. Despite of these advances in the management of febrile neutropenia empirical treatment still remains challenging due to changes in bacterial etiology [4-7]. Despite these advances in the management of febrile neutropenia, it still carries a high morbidity and mortality [8].

In our literature review, we found that most of the studies from different parts of the world evaluated the effectiveness and safety of PT with or without aminoglycosides [9-16]. Fewer studies analyzed glycopeptides plus PT combination as initial empirical therapy for treatment of febrile neutropenic episodes in febrile neutropenic cancer patients [1, 17-20]. Furthermore, the addition of VM to β-lactam antibiotic therapy would add toxicities and emergence of VM-resistant enterococci [1, 21].

In this study, we analyzed the outcome variables and the difference in rate of response between PT with and without the addition of VM.

## Patients and Methods

### Study design

Retrospective study on adult febrile neutropenic patients with solid tumor malignancies, who were treated with PT and PT plus VM at King Fahad Specialist Hospital, Dammam Saudi Arabia.

#### Inclusion criteria

Eligible patients were those with fever defined as the elevation of single oral temperature measurement of equal or greater than 38.3 °C (101 °F) or a temperature of equal or greater than 38.0 °C (100.4 °F) sustained over a 1-h period. Neutropenia was defined as absolute neutrophil count (ANC) of less than 500 cells/mm^3^ or predicted decrease below 500 cells/mm^3^ during the next 48 h. Male and female over the age of 18 years with solid tumor malignancies were included. Patients with presumed infectious cause of fever were included as high risk.

#### Exclusion criteria

Patients who have no evidence of neutropenia, history of allergic reactions to any of antimicrobial in the study, and patients who received any other antimicrobials and investigational drugs within 72 h were excluded. The study was approved by the hospital Institutional Reweive Board (IRB) committee. All the data were collected from electronic hospital information system, MedicaPlus and confirmed with review of the medical chart. All microbiology reports were based on Clinical and Laboratory Standard Institute (CLSI) guidelines.

### Classification of febrile episodes

Febrile episodes were categorized into three groups: 1) microbiologically defined infections (MDI): fever with microbial isolation; 2) clinically defined infections (CDI): defined site of infection (pneumonia, enterocolities or cellulite) but no microbiological confirmation and 3) fever of unknown origin (FUO): patients have a fever with no clinical evidence, nor microbial documented infection.

### Evaluation of response

The primary outcome was treatment success without modification or addition of other antibiotics to the initial treatment regimen within 72 h of therapy. Success was assessed after 72 h of antibacterial therapy.

#### Effectiveness of therapy

Effectiveness of therapy was defined as complete resolution of fever (reduction in temperature < 38 °C when measured orally and sustained for 48 h), clinical signs and symptoms of infection and eradication of any infectious organisms, without changes in initial assigned therapy. The bacteriological response was assessed after 72 h of treatment and overall response was assessed 7 days after the end of therapy.

#### Failure of therapy

The treatment was considered as a failure if the antimicrobial therapy was modified by the addition of other antibiotics or discontinuation of initial empirical therapy and continuation with other antibiotics during the 72 h of treatment. The failure of therapy is also defined as persistent fever in a patient with signs of clinical deterioration, microbiological evidence, and clinical progression of the presumed infection or adverse event associated with the antibiotic regimen. Death occurring within 72 h of treatment is considered as failure of both clinical and bacterial response.

#### Toxicities

The safety of therapy was evaluated by monitoring laboratory values. Nephrotoxicity and hepatotoxicity are defined as an increase in serum creatinine, transaminases, bilirubin or alkaline phosphatase by at least twice the upper normal limit [14, 22]. Hypokalemia was defined as an increase in serum potassium level > 10 mmol below the lower limit of the normal range [23].

### Statistical analysis

The primary analysis was a comparison of response rate for PT and PT + VM treatment groups. Data were analyzed by using descriptive statistical methods. Categorical variables were expressed as frequencies and percentages. The two groups were compared with Student’s *t* test for normally distributed data and not normally distributed data by Krsukal-Wallis H rank sum tests (non-parametric tests). The continuous variable values that normally and not normally distributed were expressed as median and range. Univariate logistic binary regression was used to determine the predictor variables associated with the effectiveness and safety of antibiotics in infections with febrile neutropenic cancer patients from the set of predictor variables. The significance between two groups was studied by using Wald X^2^ stat for categorical data whenever appropriate. The statistical significant variables in univariate analysis were included in the multivariate logistic regression analysis as stepwise selection was performed at a significant level of P < 0.2, to avoid any missing variables, which have strong relationship for effectiveness and safety, to determine the predictive variable for effectiveness and safety. In all analysis, an alpha level of P < 0.05 indicated statistical significance with 95% CI, whenever appropriate, calculated for the assessment of differences in response rates between PT versus PT + VM in terms of outcome and mortality. If P ≤ 0.05, there is a significant difference in response rate between the variables of two treatment groups and if P > 0.05, indicates that there is a no significant difference of outcome variables between two arms. Statistical analysis was performed by using SPSS software, version 20.0 package (statistical program for social science, version 20.0, Chicago, IL, USA).

## Results

### Characteristics of patients and type of cancers

From September 2008 to May 2013, the total of 95 febrile neutropenic patients were evaluated, and 86 febrile neutropenic patients were eligible for the study. Nine patients were excluded from the study because of modification in initial empirical therapy and protocol violation, five patients received levofloxacin, and two patients had added imipenem-cilastatin, one patient had linezolid and another patient was on meropenem plus VM which were started by the primary physician. Overall 86 febrile neutropenic patients were assessed for response to therapy, of which 60/86 (69.77%) of patients received PT and 26/86 (30.23%) were treated with PT plus VM. The two groups were assessed for the response to antibacterial therapy in terms of age, sex, clinical outcome, mortality and overall success. The median age of female and male was 49.11 and 58.11 years in PT group, whereas it was 53.45 and 47.45 in the PT + VM group (P = 0. 841).

The most common underlying malignancies were colorectal, breast and lung cancers. There were no significant differences between the two treatment groups with respect to age, sex and underlying malignancies. Fifty-seven patients develop neutropenia within less than 12 days of chemotherapy. Chemotherapy-induced (CI) neutropenic patients were higher in PT treatment groups (33 patients) than in a PT + VM treatment group. Females had higher risk of developing CI neutropenia than male. Patient’s response to therapy was higher in the PT group compared to the PT + VM group (OR: 9.818; 95% CI: 2.12 - 45.32; X^2^: 13.25; P = 0.003). The details are illustrated in Table 1.

**Table 1:** Patients Demographic, Frequency of Malignancies and Outcome of Logistic Regression Analysis of PT and PT + VM therapies

Variables	PT		PT + VM		Odds ratio (Exp(B))	95% CI	X^2^ stat (df)	P value (Wald)
	Female	Male	Female	Male				
Age, median (range)	49.11 (51)	58.11 (58)	53.45 (47)	47.45 (65)	0.997	0.964 - 1.030	0.040 (1)	0.841
Sex, gender	35 (58.33%)	25 (41.67%)	16	10	1.143	0.445 - 2.932	0.077 (1)	0.781
Malignancy type								
Colorectal Ca	11 (55%)	9 (45%)	3 (60%)	2 (40%)	Nil	Nil	26.874 (14)	0.841
Breast Ca	18 (100%)	0	4 (100%)	0				
Ca pancreas	1 (20%)	4 (80%)	0	1 (100%)				
Lung Ca	0	4 (100%)	1 (50%)	1 (50%)				
Sarcoma	1 (33.3%)	2 (66.7%)	3 (75%)	1 (25%)				
Gallbladder Ca	1 (50%)	1 (50%)	0	0				
Malignant of bile duct	0	1 (100%)	0	0				
Esophagus Ca	0	1 (100%)	0	1 (100%)				
Ovarian Ca	1 (100%)	0	1 (100%)	0				
Prostate Ca	0	2 (100%)	0	0				
Squamous cell Ca	1 (100%)	0	0	0				
Testicular Ca	0	1 (100%)	0	3 (100%)				
Nasopharynx	0	0	1 (50%)	1 (50%)				
Uterus Ca	0	0	3 (100%)	0				
Chemotherapy					9.818	2.127 - 45.321	13.258 (1)	0.003
< 12 days	21 (63.6%)	12 (36.4%)	15 (62.5%)	9 (37.5%)				
> 12 days	14 (51.9%)	13 (48.1%)	1 (50%)	1 (50%)				
Success					0.000	0.000 - 0.000	0.725 (1)	1.000
< 7 days	35 (59.3%)	24 (40.7%)	16 (59.3%)	10 (38.5%)				
> 7 days	Nil	1 (100%)	Nil	Nil				
Mortality (within sex)					NC	NC	NC	NC
Yes	Nil	Nil	Nil	Nil				
No	35 (100%)	25 (100%)	16 (100%)	10 (100%)				

NC: not computed; PT: piperacillin-tazobactam; VM: vancomycin.

### Clinical response, site of infection and microbial distribution

The distribution of infection categories did not differ considerably in the two treatment groups. Clinically documented infection occurred in 16 patients, nine (56.2%) females and seven (43.8%) males in PT group and in seven patients, four (57.1%) females, three (42.9%) males in PT + VM (P = 0.98) groups, respectively. No sign of the fever was found in 24 patients, 15 (62.5%) females, nine (37.5%) males in PT group and 10 patients, five (50%) females, five (50%) males in PT + VM groups (P = 0.89), respectively. The pleural effusion and Lung consolidation were found in 9/16 patients in PT group (P = 0.72) and 6/7 patients in PT + VM group (P = 0.19). There is no significant differences between two groups with respect to response to therapy (Table 2).

**Table 2:** Response to Therapy of PT Versus PT + VM Treatment Groups

Effectiveness variables	Baseline	PT		PT + VM		Odds ratio	95% CI	X^2^ stat (df)	P value
		Frequency (F)	Frequency (M)	Frequency (F)	Frequency (M)				
Fever	< 38.1 °C	35 (58.3%)	25 (41.7%)	16 (61.5%)	10 (38.5%)	NC	NC	NC	NC
	> 38.1 °C	Nil	Nil	Nil	Nil				
Pleural effusion	Yes	9 (56.2%)	7 (43.8%)	3 (50%)	3 (50%)	0.825	0.281 - 2.422	0.124 (1)	0.726
	No	26 (59.1%)	18 (40.9%)	13 (65%)	7 (35%)				
Consolidation	Yes	5 (14.3%)	4 (16%)	4 (25%)	3 (30%)	2.088	0.682 - 6.395	1.621 (1)	0.197
	No	30 (58.8%)	21 (41.2%)	12 (63.2%)	7 (36.8%)				

The duration of fever was longer for PT + VM group with a median of 2 days, range 1 - 5 days as compared to a median of 1 day, range 1 - 6 days in PT group. The duration of fever was slightly higher in females. However, it was noted that there was no statistical differences between the two groups (P = 0.114). Although there was no difference in the length of stay, but it was considerably higher in males, with a median of 13 days, range 1 - 38 days in the PT group (P = 0.44). Also it was noted that there was no significant differences in the duration of therapy between the two groups (P = 0.91). The duration of neutropenica was found to be higher in the female’s with a median of 6 days, range 1 - 5 days, with mean rank 54.48 in the PT + VM group compared to PT group with median, 4 days, range 1 - 10 days, mean rank 38.74 (P = 0.007) (Table 3).

**Table 3:** Overall Outcome of PT Versus PT + VM Therapies

Outcome variables	Gender	PT		PT + VM		X^2^ stat (df)	P value
		Duration (median)	Mean rank	Duration (median)	Mean rank		
Duration of fever (days), median (range)	F	2 (5)	40.93	2 (5)	49.42	0.012 (1)	0.114
	M	1 (6)		2 (3)			
Duration of neutropenia (days), median (range)	F	4 (10)	38.74	6 (5)	54.48	7.387 (1)	0.007
	M	7 (13)		7.5 (6)			
Length of stay (day), median (range)	F	7 (49)	44.85	8 (55)	40.38	0.583 (1)	0.445
	M	13 (38)		7.5 (22)			
Duration of therapy (days), median (range)	F	6 (13)	43.69	PT: 6 (7) VM: 5 (12)	43.06/13.50	0.012 (1)	0.913
	M	7 (13)		PT: 6 (11) VM: 3 (10)			

Out of 40 febrile neutropenic patients with microbiologically documented infections, 30 gram-negative pathogens were isolated from 27/60 (45%) of patients (14/27 (51.9%) were female and 13/27 (48.1%) were male) and 20 gram-positive microorganisms were isolated from 13/26 (50%) of patients (9/26 (69.2%) female and 4/26 (30.8%) male). The 60% of gram-negative isolates and 40% of gram-positive isolates were treated with PT group, whereas 60% of gram-positive and 40% of gram-negative infections were treated with PT + VM group. The types of organisms causing infection in the two treatment arms were similar, with slightly more gram-negative bacteremia. There was no statistical significance with respect to microbial eradication between the two treatment arms (OR: 1.22; 95% CI: 0.486 - 3.072; X^2^: 0.18; P = 0. 67).

Out of 30 gram-negative isolates, the common pathogens were *Escherichia coli* (n = 18), *Klebsiella pneumonia* (n = 4) and *Pseudomonas spp* (n = 3). The susceptibility to PT was 79.92%, 83.23% and 69.23% for *E. coli*, *Pseudomonas spp* and *Klebsiella pneumonia* respectively. Gram-positive pathogen susceptibility to VM was 100% for *Staphylococcus aureus* and *Streptococcus spp*, *Staphylococcus spp* 99.8% and *Enterococcus spp* 98.85%. The details of susceptibility and resistance patterns are listed in Table 4. The most common isolation sites were bloodstream followed by urine, wound, body fluids and sputum.

**Table 4:** Bacterial Spectrum, Susceptibility and Resistance Patterns of Microorganisms

Organisms	Number of isolates	%	PT		VM		OC	
			S %	R %	S %	R %	S %	R %
Gram-positive organisms								
* Staphylococcus aureus*	7	35%	-	-	100	0	71.48	28.52
* Streptococcus spp*	7	35%	-	-	100	0	69.55	30.45
* Corynebacterium spp*	3	15%	-	-	-	-	-	-
* Enterococcus spp*	2	10%	-	-	98.85	1.15	Nil	Nil
* Staphylococcus spp*	1	5%	-	-	99.8	0.2	13.73	86.27
Gram-negative organisms								
* Escherichia coli*	18	60%	79.92	20.08	-	-	-	-
* Klebsiella pneumoniae*	4	13.33%	69.23	30.77	-	-	-	-
* Pseudomonas spp*	3	10%	83.23	16.77	-	-	-	-
* Aeromonas hydrophylia*	1	3.33%	-	-	-	-	-	-
* Stenotropomonas multipholia*	1	3.33%	-	-	-	-	-	-
* Serratia marcescens*	1	3.33%	93.66	6.34	-	-	-	-
* Providencia stuartii*	1	3.33%	-	-	-	-	-	-
* Enterobacter spp*	1	3.33%	67.41	32.59	-	-	-	-

Overall, the improvement rate with or without VM in initial empirical therapy was 59/60 (98.33%) in PT and 100% in PT + VM. There is no drug-induced mortality during treatment.

There was no major difference in electrolyte imbalance, namely, magnesium and potassium. Blood urea nitrogen was elevated in PT group, but multivariate logistic regression shows that there was no significant difference between PT and PT + VM treatment groups (P = 0.114).

In this study, we developed two models, model I, which is the relationship between outcome and variables (univariate logistic regression) and model II, for determining the predictor variables for a specific outcome (multivariate logistic regression) as shown in Table 5.

**Table 5:** Logistic Regression Analysis of Variables Associated With Treatment Success Without Treatment Modification

Variables	Univariate OR (95% CI)	Univariate (P)	Multivariate OR (95% CI)	Multivariate (P)
Chemotherapy, < 12 days vs. > 12 days	9.818 (2.127 - 45.32)	0.003	3.442 (1.29 - 754.09)	0.034
Lung consolidation, yes vs. no	2.088 (0.682 - 6.395)	0.197	2.305 (1.323 - 75.003)	0.026
Blood pressure, baseline: 90/60 to 130/90 mm Hg vs. < 80/50 and > 200/120 mm Hg	0.078 (0.024 - 0.258)	0.001	-3.094 (0.005 - 0.386)	0.005
WBC, baseline: 4.5 - 11 × 10^9^/L vs. < 4.5 - 11 × 10^9^/L	0.233 (0.084 - 0.652)	0.005	-1.923 (0.021 - 1.01)	0.051
Hemoglobin, baseline: 13.5 - 17.5 g/dL vs. < 13.5 - 17.5 g/dL	0.146 (0.053 - 0.404)	0.001	-2.628 (0.011 - 0.477)	0.006
Blood urea nitrogen, baseline: 2.7 - 7.2 mmol vs. < 2.7 - > 7.2 mmol	0.267 (0.102 - 0.700)	0.007	-1.343 (0.049 - 1.382)	0.114
Serum creatinine level, baseline: F, 47 - 115/M, 53 - 88 mmol/L vs. > 47 - 115/M, > 53 - 88 mmol	3.319 (0.692 - 15.919)	0.134	-0.173 (0.046 - 15.466)	0.907
ALP, baseline: 54 - 144 mmol/L vs. > 144 mmol/L	5.111 (1.380 - 18.929)	0.015	2363 (0.571 - 193.476)	0.113
AST, baseline: 8 - 38 U/L vs. > 38 U/L	6.25 (0.768 - 50.860)	0.087	1.546 (0.051 - 432.207)	0.503
GGT, baseline: 15 - 85 U/L vs. > 85 U/L	2.539 (0.931 - 6.928)	0.069	0.422 (0.14 - 16.174)	0.726

ALP: alkaline phasphatase; AST: aspartate aminotransferase; GGT: gama-glutamyl transepiptidase.

### Model I: univariate logistic regression for predictor variable

Univariate logistic regression analysis identified 10 variables with P value of less than 0.2, which includes patients on chemotherapy, consolidation in lung, blood pressure, white blood cells (WBC), hemoglobin, blood urea nitrogen, serum creatinine level, alkaline phosphatase, aspartate aminotransferase and gama glutamyl transpeptidase. These variables were further analyzed with the multivariate logistic regression model to determine the dependent predictor variables for effectiveness of study antibiotics. Univariate logistic regression revealed that the response to PT therapy was almost OR of 9.818 more likely among subjects who develop neutropenia less than 12 days of chemotherapy regimen (P = 0.003) (95% CI: 2.12 - 45.32). Similarly, the success was almost OR of 2.088 more likely among patients who develop Lung consolidation with no significant difference (P = 0.197) (95% CI: 0.682 - 6.395), as shown in Table 5.

The elevated aspartate aminotransferase was nearly OR of 6.25 with 95% CI of 0.768 - 50.860 (0.087), increase in alkaline phosphatase was closely OR of 5.111 with 95% CI of 1.380 - 18.929 (0.015) and the increase in gama glutamyl transpeptidase level was almost OR of 2.539 with 95% CI of 0.931 - 6.928 (P = 0.069), occurring in patients treated with PT therapy. There was considerable difference in elevation of alkaline phosphatase in patients who treated with PT therapy. The increase in serum creatinine was almost OR of 3.319 with 95% CI of 0.692 - 15.919 but there is no significant difference between the two treatment groups (P = 0.134) as shown in Table 5.

### Model II: multivariate logistic regression for predictor variables

The multivariate logistic regression analysis was performed to estimate the predictor variables for effectiveness and treatment success of studied antibiotics. Among tested predictor variables in univariate analysis, the five variables were statistically significant predictors of effectiveness: blood pressure (P = 0.005), hemoglobin (P = 0.006), consolidation (P = 0.026), patients on chemotherapy (P = 0.034) and WBC (P = 0.051) as shown in Table 5.

Of these variables, the patients who develop neutropenia within 12 days of chemotherapy administration were improved with OR of 3.442 with 95% CI of 1.29 - 754.09 (P = 0.034) in PT group. The odd of treatment success with PT was OR 2.305 with 95% CI of 1.323 - 75.003 in patients who develop consolidation (P = 0.026). The success rate was higher in PT than PT + VM group, which is statistically significant.

### Toxicity

With regard to toxicity, the odds increase in alkaline phosphatase level with PT therapy was nearly 2.363 with 95% CI of 0.571 - 193.476 (P = 0.113) and with PT + VM therapy was 1.546 with 95% CI of 0.051 - 432.207 (P = 0.503), which is statistically not significant. The comparison of univariate and multivariate logistic regression outcome variables of PT vs. PT + VM treatment groups was shown in the Table 5. The increase in blood urea nitrogen was almost similar in both treatment arms which was statistically significant (P = 0.007). The multivariate logistic regression analysis shows no statistical significant with 95% CI of 0.049 - 1.382 (P = 0.114) in subjects treated with PT and PT + VM groups as shown in Table 5.

## Discussion

Over the past 40 years, the standard treatment regimen in febrile neutropenia has been changed in response to the emergence of new organisms [24]. The concept of combination therapy has been widely accepted because of their synergistic action and broad coverage of gram-positive and negative organisms [8, 25]. Many investigators support the theory of synergism of antibiotic combinations [26]. Most commonly used combination therapies, namely, an anti-pseudomonal beta lactam (PT, cefepime, ceftazidime and carbapenems) plus an aminoglycosides (amikacin, gentamycin or tobramycin) is one of the most recommended regimens in the treatment of high risk febrile neutropenic cancer patients [2, 25]. A major drawback of combination therapy is the increased likelihood of toxicities and associated costs [8, 25]. The addition of glycopeptides, VM, is not recommended as a routine part of initial therapy because it may increase the risk of complications, namely, drug toxicities, fungal super infections and therefore it should be considered carefully [5, 12]. Currently, the major issue is whether VM should be included as a part of initial therapy to cover for resistant gram-positive pathogens in the case of suspected catheter-related infections, skin and soft tissue infections and pneumonia or hemodynamic instability [5, 8, 25, 27]. European Organization for Research and Treatment of Centers (EORTC) recommends that the addition of VM to initial therapy should be considered where gram-positive organisms are predominant [5, 12, 27]. In addition, the various studies suggested that addition of VM is probably not needed as a part of initial therapy or should be stopped unless there is gram-positive organisms from blood cultures. Most of gram-positive organisms are not lethal [20, 27, 28].

The main concern of our study was to evaluate the effectiveness of PT with or without VM as an initial therapy in terms of response to therapy, mortality and drug toxicities.

In this study, the resolution of fever was observed in 86 febrile episodes, and in PT group the median duration of fever was 1 day (range, 1 - 6) as compared with 2 days (range, 1 - 5) in the PT + VM group. The overall response was similar in both therapies. There is no significant difference between the two treatment arms in terms of resolution of fever (P = 0.114). The finding in this study is consistent with previous studies [1, 11, 20, 26, 29]. The response to therapy is directly related to neutrophil count and trend [17]. The absolute neutrophil count was improved 100% (60/60) in PT group and slightly lower 96.15% (25/26) in the PT + VM group (P = 1.000). The duration of neutropenia was slightly higher in the PT + VM group than PT group. There is a significant difference in terms of median duration of neutropenia in the two treatment arms (P = 0.07). Many studies reported the duration of neutropenia for PT but fewer studies were discussed in PT + VM therapy [1, 14, 17, 24, 30]. The median length of stay was almost similar in two treatment groups. Though the length of stay was (median, 13 days) slightly higher in males with PT group but statistically there is no significant difference in two treatment arms (P = 0.445). The result is almost similar with previous studies [11, 24].

PT is highly effective against gram-positive bacteria, including β lactamase producing strains. It inhibits wide spectrum of gram-negative bacilli, including pseudomonas aerugenosa [9]. Del Favero et al 2001 reported the eradication of bacteremia due to *E. coli* was 42% in PT vs. 53% in imipenem-cilastatin. Mouton and associates performed an open, non-comparative study at 36 sites from six countries, with using PT, clinical cure occurred in 96% and bacterial eradication was noted in 93% of the cases. In the same study 15 pathogens were susceptible and all of them eradicated. In another study with PT, clinical cure rate was 74% and microbial eradication was 70%. Harter et al 2005 reported in their study that there was no difference in eradication of gram-positive pathogens between PT versus ceftazidime. Several studies from different centers have shown that PT was effective against gram-positive and negative pathogens [9, 17, 26, 29, 31]. VM should be included as a part of initial therapy in cancer institutions where gram-positive bacteria is a problem [20, 21]. In one study, the response rate of VM was 66% and another study, 100% in severe gram-positive infections in febrile neutropenic cancer patients [19]. Cometta et al 2003 in their study concluded that the empirical addition of VM therapy is of benefit for gram-positive bacterial infection that is resistant to PT; the glycopeptides had no effect on the time to defervescence, resolution of fever and all-cause mortality [1]. It is reasonable to include VM in empirical therapy where gram-positive organisms were predominant [1]. VM is a drug of choice with predictable activity against MRSA [25].

In our study, the predominance of gram-negative bacteria compared to resistant gram-positive bacteria explains the results of no added benefit of empirical VM. Overall in 86 febrile neutropenic patients, 30 isolates were gram-negative and 20 were gram-positive, and seven isolates of *Staphylococcus aureus* were methicillin-resistant (MRSA). The microbial eradication rate was 50% (27/60) in PT + VM and 45% (27/60) in PT group. Our study results were consistent with previous studies. To our knowledge, in our literature review, we did not find enough clinical studies, especially at local setting on comparison of PT versus PT + VM in febrile neutropenic patients with solid tumor malignancies.

In our study, we found no major difference in electrolyte imbalance, namely, magnesium and potassium. Blood urea nitrogen was elevated with the PT group, but multivariate regression showed no significant difference between PT and PT + VM groups (P = 0.114). Some studies reported that the electrolyte imbalance is due to aggressive chemotherapy and the hypokelamia was found only in 4% of the population receiving PT therapy [32, 33].

The majority of studies was reported that the increase in serum creatinine level was found with the addition of VM [1, 20]. Some studies reported no difference in toxicities with the addition of VM [1, 17]. The present study found that the overall success rates of therapy were similar in both the groups.

### Conclusion

PT has broad spectrum of activity against gram-positive and gram-negative infections. The addition of VM should be considered where resistant gram-positive bacterial infection are encountered, based on the local epidemiology and antibiograms. The routine addition of VM may increase toxicities without changing the clinical outomes.
